# Psychological Artificial Intelligence Service, Tess: Delivering On-demand Support to Patients and Their Caregivers: Technical Report

**DOI:** 10.7759/cureus.3972

**Published:** 2019-01-28

**Authors:** Angela Joerin, Michiel Rauws, Mary Lou Ackerman

**Affiliations:** 1 Psychology, X2AI Inc., San Francisco, USA; 2 Internal Medicine, SE Health, Markham, CAN

**Keywords:** chatbot, text-based therapy, teletherapy, employee wellness, workforce mental health, computer based therapy, remote patient monitoring, caregiver support, nurse support, healthcare technology

## Abstract

This technical report highlights how one mental health chatbot, or psychological artificial intelligence service named Tess, has been customized to deliver on-demand support for caregiving professionals, patients, and family caregivers at a non-profit organization. This low-cost, user friendly, and highly customizable service allows emotional support to be scaled to thousands of people at a single time. The following report describes the phased approach to implementing Tess in order to reach staff, caregivers, and patients across Canada and the United States.

## Introduction

Mental illness has personal, professional, and economic impacts. Research indicates that 6.8% of the workforce is directly impacted by depression and $11,936 is lost annually on average per depressed employee due to absenteeism, disability and presenteeism [1]. Barriers such as stigma and access issues prevent nearly 60% of people with a mental health problem from seeking help [2,3]. Digital solutions such as mobile and web-based applications offer an alternative source of support. One study found nearly 70% of participants expressed interest in using a digital application to self-manage their mental health [4]. Individuals who find it challenging to talk about sensitive issues may be more likely to share and disclose using mobile apps than through traditional in-person therapy [5]. Psychological artificial intelligence services and mental health chatbots have been shown to significantly reduce symptoms of depression and anxiety in just two to four weeks of interaction [6,7].

Inspired by the opportunity to help thousands of people around the world at the same time, X2AI Inc. (X2) developed the customizable mental health chatbot called Tess. Accessing support from Tess is convenient via existing communication channels such as text messaging (SMS) and Facebook Messenger, and can be integrated with Amazon Alexa/Google Home for voice-enabled services. In addition to scalability, digital solutions offer the added benefit of easily integrating with existing programs and online courses, such as the Elizz Caregiver in the Workplace program at SE Health. SE Health is a Canadian, not-for-profit and charitable organization that has, since 1908, been responding to client, family, and health system needs, primarily in the home and community. In partnership with X2, SE Health expanded their employee service offerings to include Tess and deliver 24/7 and on-demand emotionally supportive conversations [8]. This report describes the phased approach to implementing Tess in partnership with SE Health to support staff, patients, and family caregivers across Canada.

## Technical report

Phase I: Program customization and evaluation

Tess is designed using a combination of technologies, emotion algorithms, and machine learning techniques to support a variety of features. In collaboration with mental health professionals, Tess is trained to deliver pre-scripted interventions in order to replicate an empathic response that is appropriate to the inputted emotion or scenario. Specific interventions are triggered based on the individual's reported concern. If someone indicates that they feel anxious, Tess may offer a strategy to help them achieve a more relaxed state, or triage them to the appropriate resource. At the start of every conversation, Tess gives a disclaimer that: “It’s important for you to know that if you are in a crisis you should contact emergency services. You can type SOS for resources after our first chat is over. OK?” Thereafter, Tess offers resources anytime a person says SOS or reports suicidal ideation. Additionally, Tess can give a basic risk assessment and send a crisis alert via SMS to a counselor or crisis center to instruct them to take over the conversation in order to de-escalate the crisis.

For this initiative, X2 worked closely with counselors at SE Health to refine and create content that would align with the needs of caregivers. In addition to interviews with counselors, materials, expertise, and research from Elizz’s caregiver coaches were leveraged for content creation. A selection of helpful articles and worksheets from Elizz.com were identified as containing strategies to encourage self-awareness, self-care, resilience building, and burnout reduction. Tess was then trained to deliver the most meaningful elements as interventions in a conversational manner, somewhat like an interactive self-help book. To evaluate the mental health chatbot and new content, a subset of SE Health’s caregiver employees received on-demand access to Tess over a 30-day period. The purpose of caregiver interactions in this first phase was to help develop Tess’ ability to hold meaningful, relevant, and helpful conversations. Caregivers ranged from 20 to 59 years of age with the majority between 50 and 59. They identified themselves as parents, friends, grandparents, children and cousins of individuals they were providing care to.

**Figure 1:**
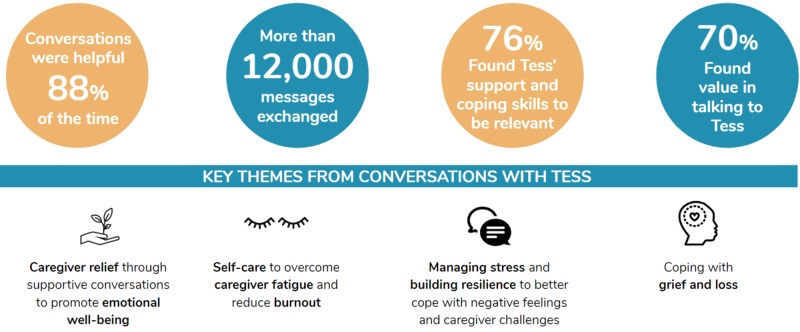
Outcomes from the preliminary launch of Tess to support caregivers

The success of this initiative was reflected in the results, which displayed high engagement with more than 12,000 messages exchanged between the caregivers and Tess. It takes approximately two minutes per message for a case manager, coach, or therapist to respond, as they need to type a response, wait for the reply, and read it. If SE Health were to deliver the same level of support without using Tess, it would cost their staff 24,000 minutes, which is equivalent to two months of work by one full-time employee when assuming an eight-hour workday, which at $65 per hour would be the equivalent of $26,000 in wages. Nearly 8% of staff actively use Tess, which equates to 675 staff members of the total 9,000 staff at SE Health. That engagement level places the equivalent savings for the entire population at approximate $585,000. Furthermore, conversations with Tess were rated as helpful 88% of the time, 70% participants have found value in having conversations with Tess, and 76% of participants found Tess provided relevant support and coping tips all or most of the time (Figure 1).

Phase II: User experience optimization and service scaling

Since the first SE Health employee interacted with Tess, the system has been expanded to include 800 different supportive interventions, which enable over 3,000,000 unique conversations. Over 50 coaches and psychologists have written the conversations for Tess. Conversations are personalized to each employee's needs to deliver a range of interventions from cognitive behavioral therapy to deeper levels of support like psychodynamic therapy interventions. Quantitative and qualitative results from the trial period were considered in order to enhance the system and improve the user experience. For example, modules that received the highest helpfulness ratings based on Tess asking “was that helpful?” after every intervention were prioritized to be delivered before others. Modules that were rated less helpful were improved by analyzing a variety of metrics that may have contributed to the helpfulness of more successful modules (i.e., module length, language, intervention themes, etc.). Additional updates included changes in outreach frequency of check-ins, and increased capacity to identify emotions, answer questions, and deliver relevant resources.

**Figure 2:**
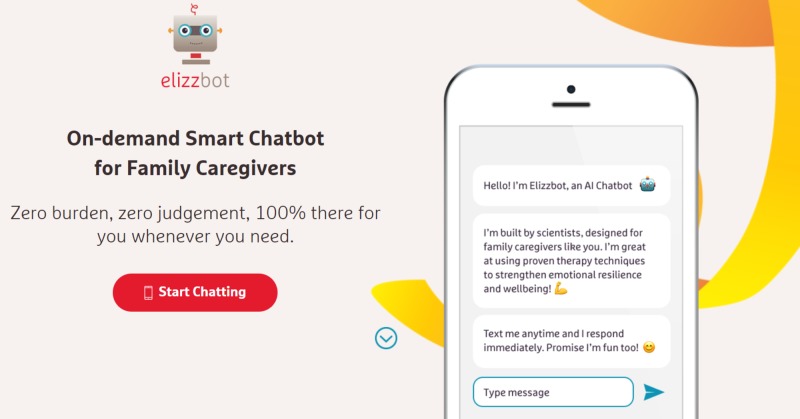
Elizzbot landing page

For the consumer version of Tess designed for family caregivers, the chatbot was customized in a number of ways and white labeled as Elizzbot (Figure 2). It was repositioned as a “helpful friend,” where the conversational copywriting was updated to reflect an encouraging and positive personality. Elizz caregiver coaches used the self-service X2 customization platform to add new relevant conversations to deal with the common emotions experienced by family caregivers. The user experience was extensively and systematically tested to optimize the conversation flow and error management. Unique elements were incorporated from a highly approachable, consumer-focused lens.

Today, the Elizzbot version of Tess offers on-demand emotional support to reduce burnout and improve wellbeing to employees at SE Health, as well as visitors on Elizz.com. Caregivers like Jillian Bohac now have access to convenient support when feeling overwhelmed. As a full-time caregiver for her husband, Jillian is reminded by Elizzbot to care for herself; she explains how “*as a social worker, you’d think I’d know better, but it sneaks up on you, the self-neglect* [9].” With a deeper understanding of the issues faced by caregivers, the system personalizes support to best meet each person’s needs and reinforces skills practiced in previous discussions with check-in’s to help build resilience.

Phase III: Extend support to patients

SE Health and X2 are preparing to expand the reach of the mental health chatbot in order to deliver more holistic support to caregivers and their patients. Through a grant awarded by the Baycrest Centre for Aging and Brain Health Innovation (CABHI), older adults are given access and able to talk to Tess through Amazon Alexa or Google Home. This version is customized to deliver voice-enabled emotional support to older adults with a focus on social isolation, loneliness, depression, and anxiety. A study with older adults is currently being prepared to show the effectiveness of the intervention. Results will support X2 in measuring the return on investment for healthcare providers and independent living facilities.

For the aging population, declining mental health has profound implications on personal, economic, and societal wellbeing. Social isolation and loneliness have been linked to increased risk of premature mortality [10], with links to coronary heart disease, stroke, and increased prevalence of dementia [11]. Understanding that loneliness in older adults is often triggered by physical and/or social losses, Tess will help this population adjust to changes and make new connections. The continuous learning of an artificial intelligence platform will offer a safe space for older adults to engage in conversations about their concerns via text or voice. The voice-enabled interface will be particularly supportive for participants with physical limitations or for those who find texting to be uncomfortable or inconvenient.

The impact of Tess on relieving feelings of social isolation and loneliness will be measured by the Duke Social Support Index (DSSI) [12]. Symptoms of depression and anxiety will also be measured before and after the Tess intervention based on scores from the Patient Health Questionnaire (PHQ-9) and Generalized Anxiety Disorder Scale (GAD-7) [13,14]. Finally, user satisfaction and feedback will be gathered directly through interactions with Tess throughout the intervention with questions such as “was that helpful?,” “what could I do to improve our conversations?” and “what have been the best/worst things about our interactions?” At least 20,000 older adults will be granted unlimited access to Tess across Canada and the United States.

## Discussion

The mental health chatbot covered in this paper offers convenient support to people in the moment they need it most through existing communication channels such as smartphones, tablets, laptops and computers. This is particularly beneficial for demographics where access to mental health support is limited in scope such as hours of service, costly treatment, and when it poses other barriers to those seeking support. Emotional support from Tess was found to decrease symptoms of depression and anxiety respectively by 13% and 18% [6], while only costing a fraction of the fee for a single therapy session. With 24/7 on-demand access, active user pricing starts at $5 per month, or per employee per month pricing at scale can become as low as $0.05 per employee per year.

The innovative capacity of this service is highlighted in the system’s capacity for customization via the X2 self-service platform. This allows any provider to customize Tess in order to deliver a higher quality of care based on unique concerns, cultural values, location, and other demographic considerations. At SE Health, this service was carefully developed, tested, and scaled to support the organization's staff, patients, and public followers across Canada and around the world.

Engaging in over 20,000 patient visits every day, SE Health’s 9,000 staff maintain a pulse on the organisation needs and help steer research and innovation initiatives. SE Health staff have been central to the evolution of the Tess service into the friendly, supportive, and continuously learning chatbot known as Elizzbot. With evidence of Elizzbot’s capacity to support caregivers, continued research efforts aim to strengthen the emerging body of knowledge outlining to the use of psychological artificial intelligence to help employees, patients, and the public to overcome many of the traditional barriers to care.

(Visit Elizz.com/Elizzbot to try Elizzbot, and elizz5lifestages.com to learn more about the Elizz 5 LifeStages of Caregiving Employee Program)

## Conclusions

There is evidence that using psychological artificial intelligence to provide customized support for caregiving professionals, patients, and family caregiver is a feasible service delivery method. This report suggests that the Tess service may offer an affordable and scalable solution that accommodates the busy schedules of caregivers while helping them reduce burnout and improve resilience. Furthermore, Tess’ capacity to expand support to patients further reduces the caregiver burden and has the potential to relieve feelings of depression, anxiety, and loneliness.
